# Biological Oscillators in Nanonetworks—Opportunities and Challenges

**DOI:** 10.3390/s18051544

**Published:** 2018-05-13

**Authors:** Ethungshan Shitiri, Athanasios V. Vasilakos, Ho-Shin Cho

**Affiliations:** 1 School of Electronics, Kyungpook National University, Daegu 41566, Korea; ethungshan@ee.knu.ac.kr; 2 Department of Computer Science, Electrical and Space Engineering, Lulea University of Technology, 93187 Lulea, Sweden; athanasios.vasilakos@ltu.se

**Keywords:** nanonetworks, molecular communication, biological oscillators, clocks, survey

## Abstract

One of the major issues in molecular communication-based nanonetworks is the provision and maintenance of a common time knowledge. To stay true to the definition of molecular communication, biological oscillators are the potential solutions to achieve that goal as they generate oscillations through periodic fluctuations in the concentrations of molecules. Through the lens of a communication systems engineer, the scope of this survey is to explicitly classify, for the first time, existing biological oscillators based on whether they are found in nature or not, to discuss, in a tutorial fashion, the main principles that govern the oscillations in each oscillator, and to analyze oscillator parameters that are most relevant to communication engineer researchers. In addition, the survey highlights and addresses the key open research issues pertaining to several physical aspects of the oscillators and the adoption and implementation of the oscillators to nanonetworks. Moreover, key research directions are discussed.

## 1. Introduction

Nanomachines are to a nanonetwork what transceivers are to a cellular network or what sensors are to a wireless sensor network. In other words, they are considered to be the functional units for the new and emergent nanonetwork [1]. In a nanonetwork, the communication between the nanomachines is envisioned to occur through either the traditional electromagnetic communication [2] or the more recent molecular communication [3]. On one hand, the traditional method is a well-established method for terrestrial environments. However, the attenuation and losses that the electromagnetic waves suffers in a fluidic molecular environment makes its application seem bleak [4]. Nonetheless, the electromagnetic communication in the terahertz band are being investigated as a viable radio wave technology for non-fluidic, nanoscale communications [5]. Molecular communication, on the other hand, is favorable, as it already exists in fluidic environments. For example, signal exchanges between neurons [6], cell division [7], and metabolic signals [8] are different forms of molecular communication. Naturally, molecular communication is readily compatible for fluidic, nanoscale communications [9]. In this study, we strictly consider the molecular communication-based nanonetwork. Besides the duo, other communication techniques envisioned for nanonetworks (but less researched upon) are acoustic and nanomechanical communications [1,2]. Unless specified, the term “nanonetwork” in this article refers to a molecular communication-based nanonetwork.

From a communication systems engineer’s perspective, it would be desirable for nanomachines to have components and modules similar in function to those presently used in communication transceivers. A good illustration of such nanomachine architecture can be found in [1]. Currently, molecular nanomachines are restricted to simple tasks and are therefore characterized by low power, basic functionality, and limited capability [9,10,11]. Simple tasks include generating a response (e.g., fluorescence) upon sensing a target (e.g., toxic chemical) [12] or delivering a package (e.g., drugs) to a target site (e.g., infected area) [13]. Such tasks, however, may sometimes heavily rely on an external controller (humans or a more powerful device). For instance, in targeted drug delivery [11], the external controller has to routinely inject the drug with high precision so that the surrounding healthy tissues are not affected. Nanonetworks can leverage targeted drug delivery provided the nanomachines can be programmed to either release the drug periodically after a certain period or when a behavioral change in the target is sensed. In both scenarios, the nanonetwork has to know “when to” release the drug. The former requires the knowledge of the duration between each release and the latter requires knowledge on the precise time required for the behavioral change to communicate to the network for a coordinated release. Common to both, a “time keeper” is required [11].

### 1.1. Motivation

Several reasons drive the motivation of this study and are listed below:

- *Need for a biologically compatible time keeper:* Quartz crystal oscillators provide and maintain the time information in almost every electronic device [14]. To stay true to the definition of molecular communication, however, it would be meaningful to integrate oscillators that are made with biological components (e.g., molecules) and are driven by biochemical processes (e.g., gene translation and transcription) or, in other words, oscillators that are biocompatible [15]. Fortunately, to begin with, nature has an abundance of such oscillators. The sleep-wake cycle driven by the circadian oscillator [16], cell division controlled by mitotic oscillators [17,18], and the periodical break-down of glucose to sugar that is maintained by glycolytic oscillators [19] are few examples of biological oscillators in nature. In this study, we refer to them as *natural oscillators*. For many decades, biologists, physicists, and mathematicians have extensively studied natural oscillators, mainly to understand the underlying principles of the oscillators, which, as we will see in Section 2.1, are of a very complex nature, even though we outline only the main principles.
- *Avenues for developing a simpler system:* While the understanding of the complex mechanism that drives natural oscillators is a challenge, to engineer such mechanisms was another challenge until the birth of a field called synthetic biology [20]. Ironically, the successful realization of the first in-vivo, artificially-realized oscillator, namely, the repressilator [21] represented the beginning of synthetic biology. The repressilator laid the path for other novel designs to follow [22,23]. In this study, we refer to them as *synthetic oscillators*. Synthetic biology offers several advantages to nanonetworks. Firstly, it has led to the development of oscillators that involve much simpler mechanisms than their natural counterparts [24] and such oscillators could be embedded within a nanomachine. Secondly, such an engineering feat is a benefit to nanonetwork applications that are targeted towards living tissues, where biocompatible components that can be biologically engineered are preferred. Although these systems are still far from perfection, recent studies have shown that they can indeed be improved [25,26,27,28].
- *Investigations from a communication systems engineer perspective:* Nearing two decades since the inception of nanonetworks, few studies on oscillators in the literature have surfaced from the nanonetwork research community [29,30,31]. Taking cues from nature, these studies have presented oscillatory systems that will be suitable, in particular, for molecular nanomachines and, in general, for a nanonetwork. The first two oscillator systems were designed to allow a nanonetwork to achieve synchronization by converging the period of oscillations [29,30], while the third system was designed to align the clock times and extend the purpose of the oscillator beyond synchronization, to provide timing information for scheduling channel access and decoding the signals or for coordinating other communication modules in a nanomachine [31]. We will present, for the first time, qualitative comparisons between them.
- *Lack of a consolidated study:* To date, a consolidated literature that brings biological oscillators under one single study is lacking. Motivated by the gaps in the literature regarding biological oscillators, more specifically to nanonetworks, we provide a comprehensive review of biological oscillators from the earliest to the latest developments. Additionally, unlike other recent surveys [32], we study each oscillator using parameters that are significant in the eye of a communication systems engineer.

On a side note, readers are encouraged to refer the literature [33] for detailed explanations and visuals of the chemical reactions of the natural oscillators, supported by a rich background on historical facts and significances. Moreover, for the sake of brevity, we leave out the mathematical expressions for both the natural and synthtic oscillators as these can be found in much detail in the literature, particularly, in ref. [32].

### 1.2. Main Contributions

Based on the aforementioned rationales, the main goal of this survey is to introduce biological oscillators, in a tutorial fashion, to the nanonetwork research community and additionally, to act as a small window into the complex and intriguing world of biological oscillators to communication system engineers. The main contributions of this paper are summarized as follows:

- Consolidating the biological oscillators into a single work, which, to the best of our knowledge, no work has ever done, making this survey the first one.
- Classification of the biological oscillators based on whether they are found in nature or not.
- Reviewing the natural oscillators and their underlying mechanisms with sufficient detail, bearing in mind that not all researchers working in nanonetworks have biology backgrounds.
- Reviewing the synthetic oscillators and their design principles and properties, supported with simple and accurate visuals of the system’s schematics, bearing in mind that not all researchers working in nanonetworks have synthetic biology backgrounds.
- Reviewing the recent works on oscillatory systems proposed by the nanonetwork research community.
- Comparative analysis of the oscillators.
- Identification of open research issues for both the physical and communication aspects of the oscillators.

The remainder of this paper is organized as follows: In Section 2, we discuss the natural and synthetic oscillators in terms of their working mechanisms. Substantiating each oscillator, figures illustrating each oscillator’s oscillations are also provided. Section 3 highlights the current research issues in the physical aspects, such as molecular noise, design, and sustainability and in the communication aspects, such as adoption and implementation. The tradeoffs and future research directions are also presented. Finally, Section 4 concludes the paper.

## 2. Biological Oscillators

Any biological system, wherein there exists a source of excitation, a restorative process, and a delay element, with appropriate system parameters that lead to a cyclic behavior, can be regarded as a biological oscillator [34]. This section enumerates on biological oscillators that we believe are of significant interest to the nanonetwork research community. The section is divided into three subsections—the first subsection is dedicated to natural oscillators, the second subsection is dedicated to synthetic oscillators, and the third subsection is dedicated to synthetic oscillators that have been proposed by the nanonetwork research community.

**Figure 1:**
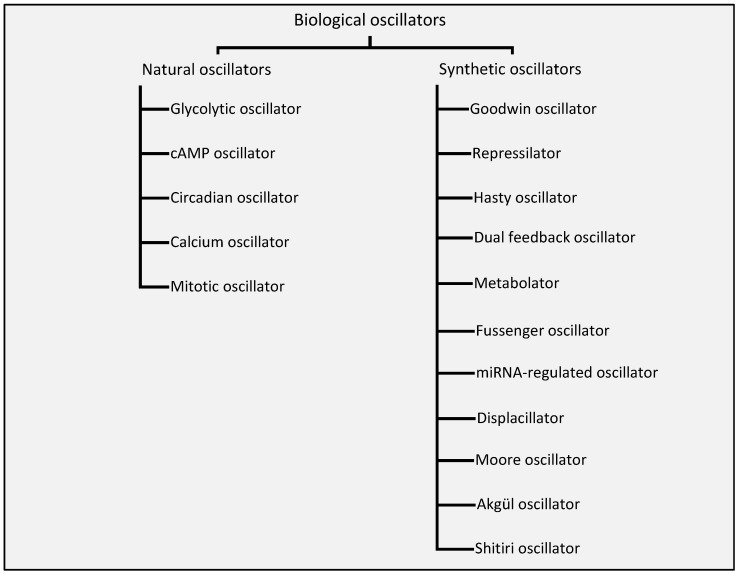
Classification of biological oscillators.

We explicitly classify, for what we believe is the first time, the biological oscillators into two broad categories depending on their existence in nature. Figure 1 shows the classification of the biological oscillators into natural and synthetic oscillators.

### 2.1. Natural Oscillators

Natural oscillators are those that exist in nature and their primary function is to regulate various processes and activities in living beings. Here, we review a list of such oscillators that have been extensively studied and are of significant importance to the field of biology.

#### 2.1.1. Glycolytic Oscillators

Glycolytic oscillators produce periodic fluctuations in the concentration of the molecules (metabolites) that are involved in the process of glycolysis [35,36]. The oscillatory phenomenon was first reported in 1957 by Duysens and Amesz [37] who were conducting studies to understand the process of glycolysis. Glycolysis is considered to be the most ancient and powerful bioenergetics (the study of energy transductions in living organisms [38]) process prevailing in all living beings [19], from bacteria to mammals, and is the most studied control system (see [38], supplementary materials). Although first reported by Duysens and Amesz, the experimental work by Betz and Chance is, however, regarded to be the first work conducted specifically on glycolytic oscillators [35].

Of the many studies undertaken to identify the main molecule that causes the oscillations [39,40,41,42,43,44], the enzyme phosphofructokinase (PFK) has been generally found to be the dominant factor. How PFK is considered the dominant factor may be attributed to its different conformational responses to adenosine triphosphate (ATP) and adenosine diphosphate (ADP). This particular behavior makes PFK an allosteric enzyme as it is allosterically inhibited by ATP and activated by ADP. Allosteric is the property by which the behavior of the enzyme is affected by a molecule binding to a specific part of the enzyme [38].

**Figure 2:**
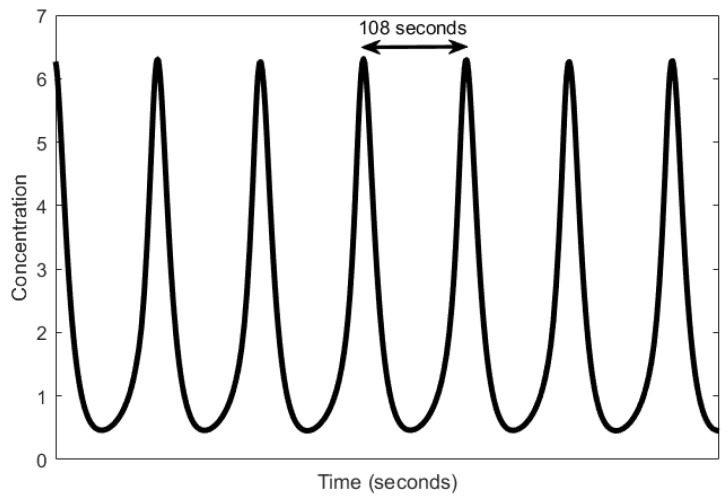
Oscillations produced in the phosphofructokinase (PFK) concentrations by a glycolytic oscillator [41].

In total, glycolysis involves 11 intermediary steps [45], all of which involve an oscillatory regime. We, however, outline here the simplified model of glycolysis as reviewed in [45], which involves PFK. When PFK is in a state called the active state, it catalyzes the phosphorylation of fructose-6-phosphate to fructose-1,6-bisphosphate using ATP [46]. During this phosphorylation process, ATP is converted to ADP and causes a rise in the level of ADP and a decline in the level of ATP. ADP is then converted back to ATP by adenosine monophosphate (AMP) and consequently, reverses the levels of ADP and ATP. Interestingly, for every ATP consumed, two ATPs are produced. The net increase in ATP levels forces PFK to become inactive, inhibiting it from catalyzing the phosphorylation. PFK resumes its action when AMP, which is present in small amounts, removes the inhibition by ATP. Thus, the cycle is reset. The period of the oscillation range is in the order of minutes, from 1.8 min (as shown in Figure 2) to 8.6 min [33]. Recent works [47,48] have demonstrated that sustained oscillations can occur in a single cell whereas they were previously thought to be generated through a population [49].

#### 2.1.2. Cyclic Adenosine Monophosphate (cAMP) Oscillator

The cyclic adenosine monophosphate (cAMP) oscillator produces oscillatory behavior through the cyclic synthesis of cAMP [33,50]. The oscillation formed is in fact a mode of intercellular communication which is found among the Dictyostelium discoideum amoeba species [51,52,53]. The amoeba forages for food either as an independent cell or as a group of cells, depending on the availability or scarcity of food, respectively [54,55,56]. To transit to the group phase during times of food scarcity, a periodic mechanism of intercellular communication is mediated by periodic secretion of the secondary messenger cAMP and leads to oscillations [33,51,55,56,57].

**Figure 3:**
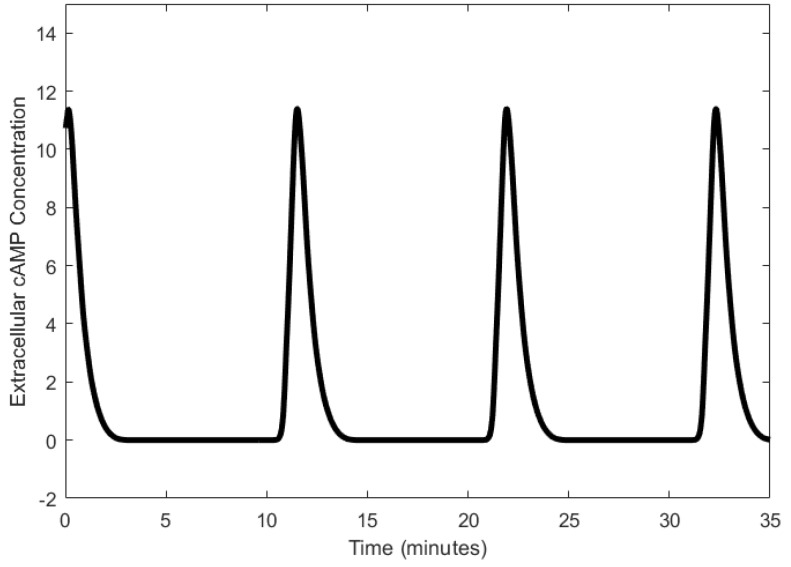
Oscillations of extracellular cyclic adenosine monophosphate (cAMP) generated using the model in [60].

We describe the operation of the cAMP oscillator as described in [33]. Adenylate cyclase, which synthesizes intracellular cAMP from ATP, is activated when extracellular cAMP binds to a receptor of the cell [58,59]. The secretion of intracellular cAMP out of the cell follows its synthesis; cAMP binds to its receptor and further promotes its own synthesis [60]. The accumulation of cAMP is generally considered to be regulated through the desensitization of the cAMP receptors when cAMP binds to them, which can be reversed through phosphorylation [61,62]. Once the receptors return to the sensitive state, cAMP can bind to them, and the synthesis of cAMP is restarted, thereby forming the cAMP oscillations. Another form of cAMP regulation is the degradation of cAMP through phosphodiesterase [63,64]. The temporal changes in the concentration of external cAMP cause relaxation oscillations with periods in the order of tens of minutes, as shown in Figure 3. 

#### 2.1.3. Circadian Oscillator

The circadian oscillator refers to an internal biological oscillator that has a free-running period of about 24 h (an oscillator with a ~24 h period but one that does not follow the day-night cycle). The term circadian, coined by Franz Halberg in 1959, 230 years after the classic experiment of 1729 that demonstrated that even in the absence of sunlight, the daily movements of the leaves of the Mimosa plant were maintained [65], loosely translates to “about a day”. Sleep-wake cycles are a good example of circadian oscillators [16]. A pivotal study in the understanding of the circadian oscillators was the identification of the *per* (period) gene [66] that expresses the PER protein in a periodic manner, leading to oscillations [67,68,69,70].

**Figure 4:**
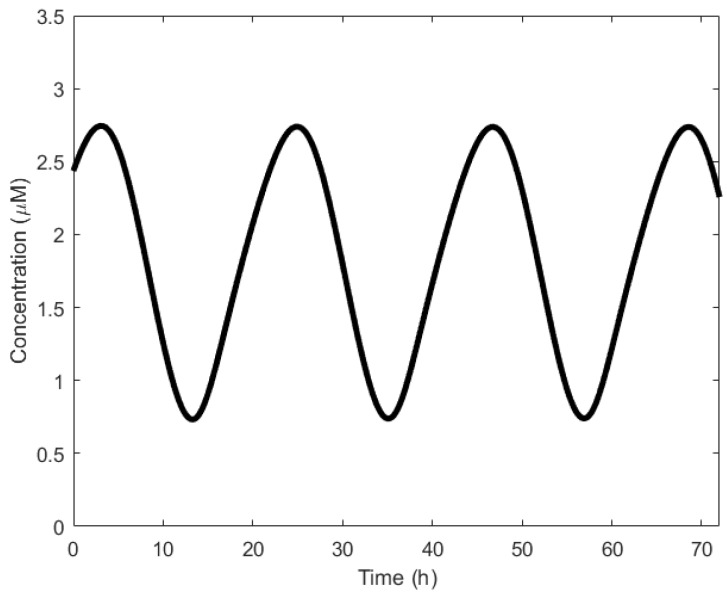
Circadian oscillations formed by the *per* gene with Goldbeter’s model [33,71].

To understand the operation of circadian oscillation, we discuss Goldbeter’s model [71]. It begins with the transportation of the *per* messenger ribonucleic acid (mRNA) from the nucleus to the cytosol [56]. The mRNA is then translated into the PER protein, causing a rise in the PER level. This PER is said to be in the inactive state. Over time, PER undergoes phosphorylation [72] and is transformed to the active state. Once in the active state, it is transported back to the nucleus where it begins to represses the transcription of the *per* gene [73]. PER, therefore, inhibits its own transcription. The cycle resumes when the PER levels in the nucleus are too low and the inhibition is removed. Figure 4 shows the oscillatory behavior of the *per* gene. Period of oscillations are generally in the order of hours.

#### 2.1.4. Calcium Oscillator

The calcium (Ca^2+^) oscillator generates periodic temporal fluctuations in the concentration levels of Ca^2+^ that arise either from the entry of external calcium into a cell or from the periodic release of intracellular calcium to the cytosol from internal stores [74]. The former is commonly found in excitable cells while the latter are found in non-excitable cells [75]. The first observations of the calcium oscillator were made in 1985 by Cuthbertson and Cobbold in fertilized mouse oocytes [76] and in hepatocytes in 1986 and 1987 by Woods et al. [77,78]. Over the years, several models have been developed to describe the mechanisms and dynamics of the Ca^2+^ oscillations. For example, while some models consider Ca^2+^ oscillations with a constant inositol 1,4,5-triphosphate (IP_3_) concentration [79,80,81,82], other models consider Ca^2+^ oscillations with a varying IP_3_ [83,84,85,86].

**Figure 5:**
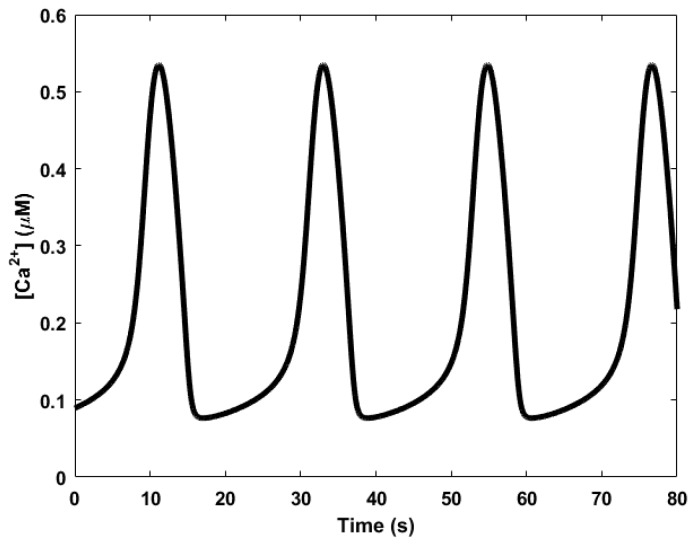
Calcium oscillations generated based on mathematical models by De-Young and Keizer [79] and Li and Rinzel [80].

Here, we review the workings of the oscillator as presented by Höfer et al. [85]. It involves the interplay between Ca^2+^, inositol trisphosphate (IP_3_), and the biphasic IP_3_ receptors (IP_3_Rs). The IP_3_ molecules bind to the biphasic IP_3_ receptors (IP_3_Rs) located on the membrane of the endoplasmic reticulum (ER)—the internal storage site for Ca^2+^ ions. The binding allows the IP_3_Rs, which act like a gate, to open up, releasing the Ca^2+^ ions stored inside the ER, thereby increasing the levels of cytosolic Ca^2+^. Two processes follow. First, when the cytosolic Ca^2+^ level is sufficiently high, the Ca^2+^ ions begin to inhibit the IP_3_Rs, effectively inhibiting the release of Ca^2+^ from the ER and consequently reducing cytosolic Ca^2+^. In addition, simultaneously, the majority of the cytosolic Ca^2+^ are pumped backed into the ER through natural pump-like structures on the ER called Na^+^-Ca^2+^ exchangers, adding to the reduction of cytosolic Ca^2+^. Second, the cytosolic Ca^2+^ triggers the regeneration of IP_3_ through PLC_δ_, which is also a secondary messenger, setting the oscillator up for the next cycle. Calcium oscillations can have periods in the order of seconds, shown in Figure 5, to several minutes.

#### 2.1.5. Mitotic Oscillators

Mitotic oscillators generate oscillations through periodic fluctuations in the level of cdc2 kinase, a cell cycle regulator. Mitotic oscillators regulate the process of cell division, namely mitosis [87]. More specifically, they determine the onset of the cell division [88]. Walther Flemming discovered mitosis in 1878 [89]. Since then, the idea that a biochemical oscillator may be in control of the onset of mitosis had long been speculated [90,91]. This speculation was validated in experiments, where cell division in the slime mold, Physarum, occurred with periods of 12 h [92,93].

**Figure 6:**
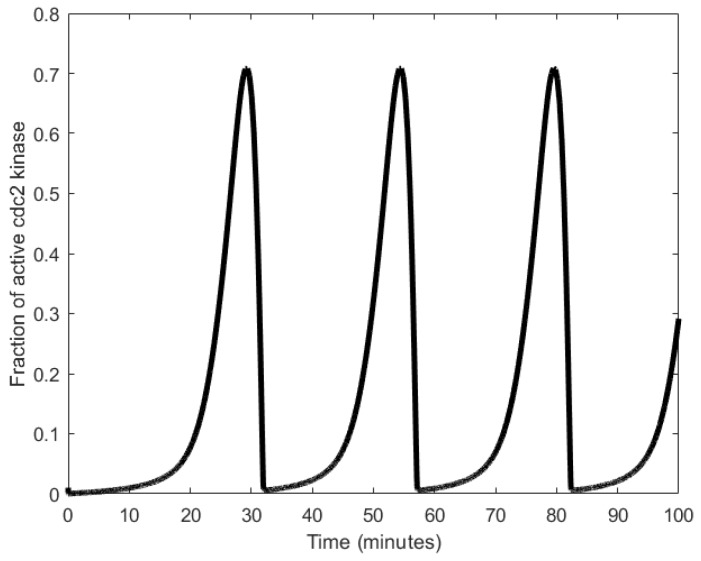
Oscillations generated from the minimal cascade model illustrating the temporal fluctuations in the concertation of cyclin [94].

To explain the workings of a mitotic oscillator, we describe here the minimal model for a mitotic oscillator [94]. A protein (cyclin) is synthesized at a constant rate to generate an enzyme (cdc2 kinase), whose active form produces a substance that degrades cyclin, resetting the oscillator [17,95,96]. More specifically, cyclin activation of the cdc2 kinase causes the transformation of cdc2 kinase from the inactive, tyrosine-phosphorylated form, denoted as M+, into the active, dephosphorylated form, denoted as M [88,94]. A negative feedback loop is created when M triggers the activation of a protease that specifically degrades cyclin. Mitotic oscillation periods have been observed to be generally in the order of tens of minutes (as shown in Figure 6), but can range to the order of hours as well [33].

Natural oscillators could provide the necessary timing information to a nanonetwork in a broadcasting manner without having to be embedded within a nanomachine. This is a key advantage as it would allow nanomachines to simply sense and extract the timing information from the ubiquitous natural oscillators. Applications such as the periodic release of drugs can monitor the periodic changes in the oscillations and time their release accordingly. However, each nanomachine will be required to be in close proximity to the oscillator and/or require an additional interface to read the oscillations. In Table 1, we summarize the characteristics of the natural oscillators and list them in the increasing order of oscillation frequency.

### 2.2. Synthetic Oscillators

Synthetic oscillators are the oscillatory systems that are developed in a laboratory setting in the field of synthetic biology. Regarded as the intersection of protein and genetic engineering with systems biology, the basic idea of synthetic biology is to engineer an artificial gene circuit that can be inserted into a host cell to perform new tasks [97]. As mentioned earlier, the realization of the repressilator [21] (and the toggle switch [98]) is said to have sparked the beginning of synthetic biology [20,99]. For an in-depth introduction to synthetic biology, readers are directed to literature [97]. We review here a set of synthetic oscillators that are significantly important, including the first ever theoretical model, which is considered to be a critical step in the design of the synthetic oscillators we know of today.

#### 2.2.1. Goodwin Oscillator

**Figure 7:**
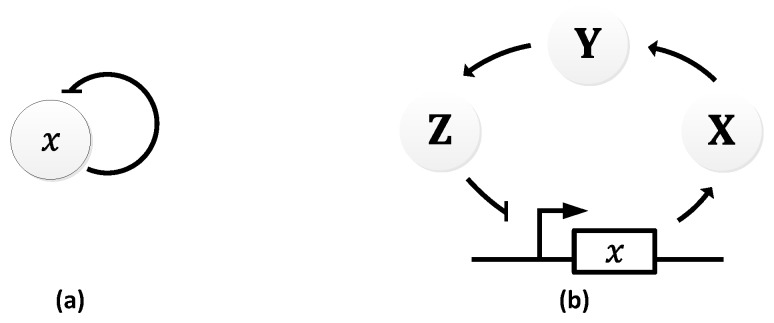
Goodwin Oscillator: (**a**) symbolic representation; (**b**) schematics with intermediary steps illustrating the self-negative feedback loop.

In 1963, B.C. Goodwin proposed a model to capture the oscillatory behavior in genetic regulatory networks [100,101]. In his model, a single gene, 𝑥, periodically represses itself through a self-negative feedback loop leading to oscillations (Figure 7a). Specifically, gene 𝑥 expresses (transcribes) a messenger RNA, X, which is translated into an enzyme, Y, which in turn catalyzes the production of a metabolite, Z, which causes the inhibition of the expression of X [56,100] (Figure 7b).

**Figure 8:**
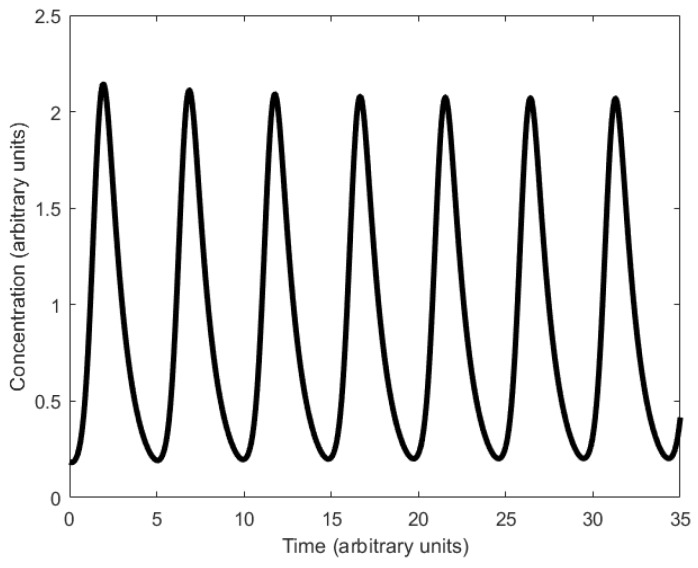
Oscillations formed by temporal concentration changes in mRNA(X) by the Goodwin oscillator.

In principle, the Goodwin oscillator can generate sustained oscillations (Figure 8), yet it requires an unrealistically large Hill coefficient value (n > 8) [102]. Nonetheless, it is the first model that has demonstrated how sustained oscillations could be obtained through a self-negative feedback loop [56] and has undoubtedly laid the groundwork for other models to follow suit. In addition, this type of negative feedback loop was later discovered in the circadian oscillators of Drosophila melanogaster and Neurospora crassa [68,103,104], leveraging the work of Goodwin.

Although no wet lab experiments were carried out, Goodwin remarked that the period of oscillation would be between 4–8 h [104]. However, in a recent wet lab experiment carried out by Stricker et al. [22], periods that were under an hour, averaging 30 min, were observed, (see [22], supplementary materials), far less than what Goodwin had remarked. The Goodwin oscillator also showed consistency in period to isopropyl β-D-1-thiogalactopyranoside (IPTG), a substance that can inhibit the repressor [22]. A far more realistic model of the Goodwin oscillator was realized by Smith [105], wherein a time delay was included in the negative feedback loop, mitigating the need for the unrealistic Hill’s coefficient value.

#### 2.2.2. Repressilator

**Figure 9:**
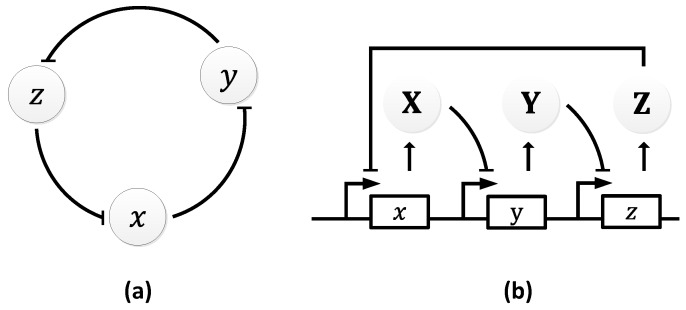
Repressilator: (**a**) symbolic representation; (**b**) schematic representation depicting the paths of the proteins, X, Y, and Z, responsible for causing repression of genes *y*, *z*, and *x*, respectively.

The repressilator [21] was the first synthetic gene oscillator to be successfully engineered. It consists of three genes, 𝑥, 𝑦, and 𝑧, that can repress each other forming a negative feedback loop (Figure 9a), making it a multi-gene variant of the Goodwin oscillator [32]. The loop begins with the first gene, 𝑥, which performs the translation of the repressor protein, X. X inhibits the transcription of the second repressor gene, 𝑦, whose protein product, Y, in turn, inhibits the expression of a third gene, z. Finally, the protein product of 𝑧, Z, inhibits the first gene, 𝑥, from performing X translation, completing the loop [21]. Figure 9b illustrates the negative feedback process.

**Figure 10:**
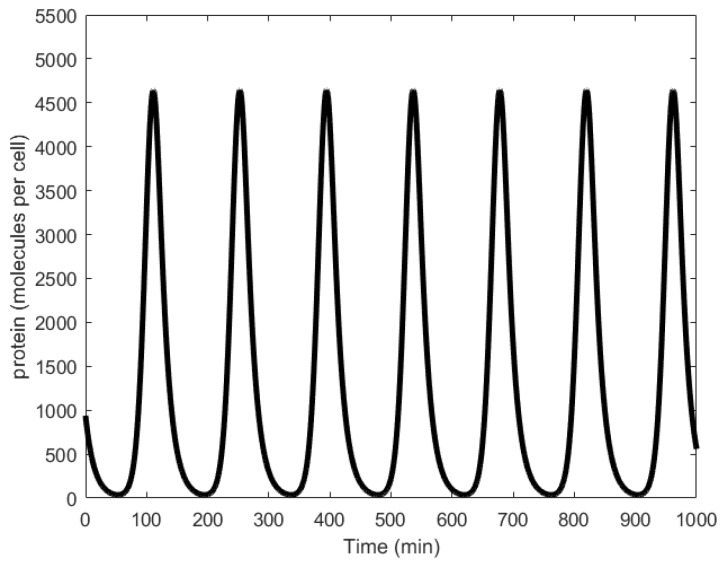
Oscillations formed by the temporal fluctuations in the concentration of protein X.

Oscillations generated through simulations were found to have average periods of 150 min (Figure 10), while wet lab experiments showed periods in the range of 160 ± 40 min with at least 40% of the engineered cells reported to generate oscillations successfully [21]. Further, significant variation in the oscillation amplitudes was observed (Figure 2c in [21]).

#### 2.2.3. Atkinson Oscillator

**Figure 11:**
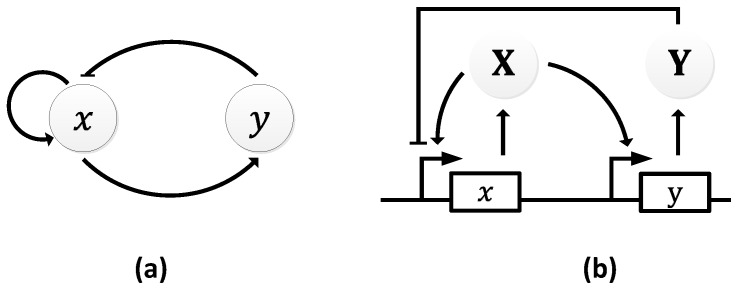
Atkinson oscillator: (**a**) symbolic representation; (**b**) schematic representation depicting the internal pathways.

The Atkinson oscillator consists of two genes, 𝑥 and 𝑦, where gene 𝑥 expresses both its own transcription and that of gene 𝑦 (Figure 11a) [106]. Specifically, gene 𝑥 expresses X, which, in turn, promotes its own expression and the expression of Y through gene 𝑦. As the level of Y rises, it begins to inhibit the transcription of the first gene causing a decline in the level of X, which resets the oscillator and a new cycle begins (Figure 11b).

**Figure 12:**
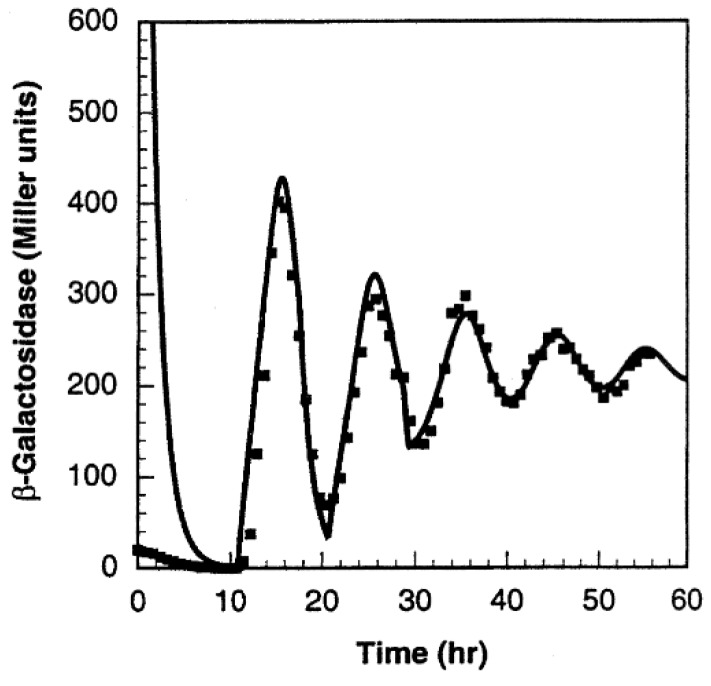
Damped oscillations formed by the Atkinson oscillator. The solid line represents the simulated results and the squares represent the wet lab experiment results [106]. Reprinted from Cell, 113, Mariette R. Atkinson, Michael A. Savageau, Jesse T. Myers, Alexander J. Ninfa, Development of Genetic Circuitry Exhibiting Toggle Switch or Oscillatory Behavior in Escherichia coli, 11, Copyright (2018), with permission from Elsevier.

In both the simulations and wet lab experiments, the period of the oscillations remains the same ~10 h (Figure 12), suggesting very good agreement between the theoretical simulation and the experiments. However, the oscillations formed were damped owing to the oscillator’s design [106]. In particular, the authors postulated that the damping was caused either by the longer lifetime of the activator, X, or by the shorter lifetime of the repressor gene, 𝑦.

#### 2.2.4. Hasty Oscillator

**Figure 13:**
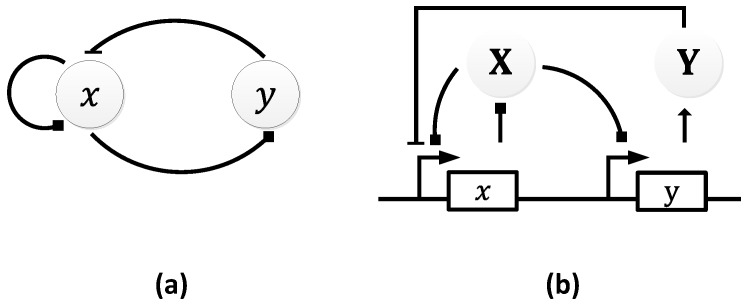
Hasty oscillator: (**a**) symbolic representation; (**b**) schematic representation elucidating the intermediary steps. Lines with squared heads indicate the variable links.

The Hasty oscillator [107] consists of two genes, one of which exhibits a switch-like behavior depending on its protein product concentration level. Gene 𝑥 expresses X at low concentrations of X and does not express X at high concentrations of X giving it switch-like behavior (see Figure 13a). The Hasty oscillator is therefore also referred to as the variable link oscillator [32]. At low concentrations of protein X, gene 𝑥 expresses X and triggers the expression of protease Y through gene 𝑦. As the concentration of X increases, as well as at some high concentrations of X, X represses its own transcription and that of Y. Protease Y degrades X, further adding to the decline in the concentration of X. Thus, in this manner, the concentration of X is regulated, resulting in oscillations. Figure 13b illustrates the oscillator in more detail.

**Figure 14:**
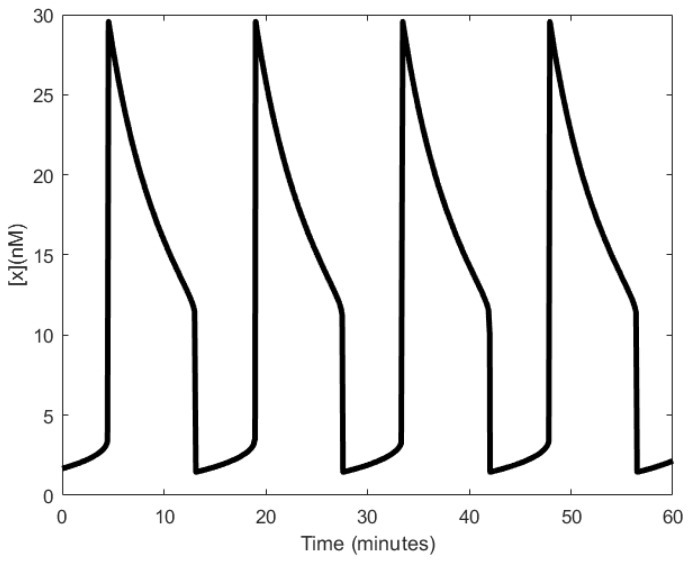
Oscillations observed by the Hasty’s oscillator.

Unlike the previous oscillators, the Hasty oscillator generates oscillations resembling a relaxation oscillator (Figure 14). Although not shown here, the average period of the oscillations ranges between 8–44 min when driven by another process that exhibits a oscillating behavior [107]. To the best of our knowledge, no wet lab experiments have been carried out to verify the model.

#### 2.2.5. Metabolator

**Figure 15:**
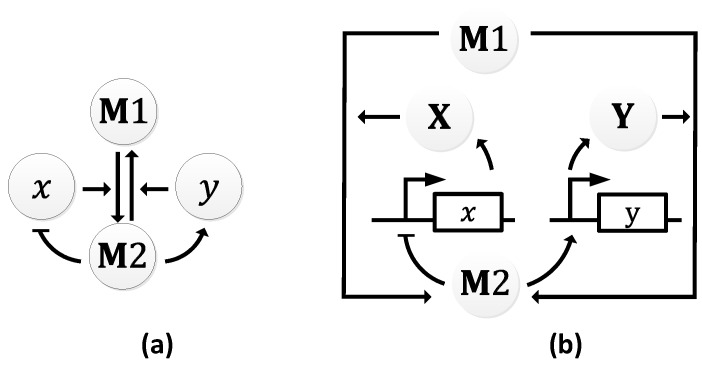
Metabolator: (**a**) symbolic representation; (**b**) schematic representation illustrating the signaling pathways.

The metabolator, as the name suggest, is a synthetic oscillator that generate oscillations in metabolites through the integration of metabolism and transcription regulation [108]. It was constructed to show that metabolic flux could control system-wide oscillations. The metabolator was conceived to consist of a flux-carrying network with two interconverting metabolite pools (M1 and M2) that are catalyzed by two enzymes (X and Y). In turn, M2 is responsible for the expression of the two enzymes. The expression of X is inhibited by M2, and the expression of Y is promoted by M2 (Figure 15a).

**Figure 16:**
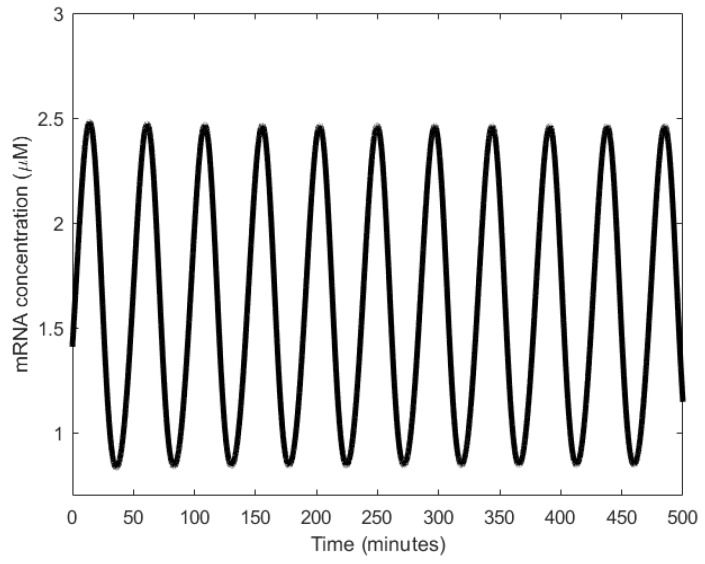
Oscillations observed in the metabolator.

At low concentration levels of M2, gene 𝑥 is not inhibited from expressing X, while gene 𝑦 is not promoted to express Y. Since X catalyzes M1 to M2, the concentration of M2 increases which results in the decline of the concentration of M1. As the concentration of M2 rises, gene 𝑥 is inhibited from expressing X, while gene 𝑦 is promoted to express Y. With the increase in the concentration of Y, M2 is converted back to M1 (Figure 15b). The resultant effect is simultaneous rise and decline in the concentrations of M1 and M2, respectively, bringing the metabolator back to the initial stage. Periods averaged about 40 min in simulations (as shown in Figure 16) and 45 ± 10 min in wet lab experiments with 60% of the engineered cells showing oscillations [108].

#### 2.2.6. Dual Feedback Oscillator

**Figure 17:**
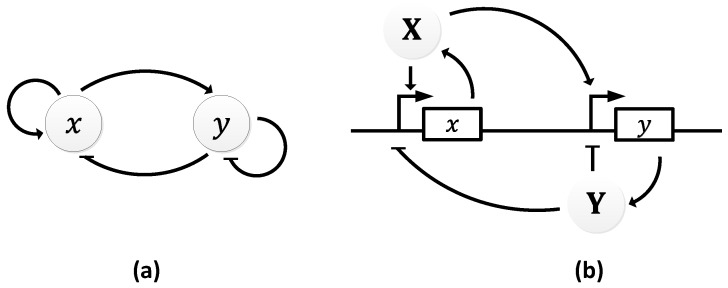
Dual feedback oscillator: (**a**) symbolic representation (**b**) schematics depicting the intermediate positive and negative loops through the proteins X and Y.

The dual feedback oscillator consist of two genes, 𝑥 and 𝑦, that are opposite in nature [22]. The oscillator is based on the theoretical model [109] but with a common promoter [110]. Gene 𝑥 promotes its own transcription as well as that of 𝑦 (positive feedback loop), while 𝑦 represses its own transcription as well as that of 𝑥 (negative feedback loop) (Figure 17a).

Proteins X and Y, respectively, mediate promotion and repression in the following manner. Firstly, the transcription of protein X by gene 𝑥 occurs. X can express both 𝑥 and 𝑦 simultaneously, thereby increasing their concentrations. Concurrently, protein Y is expressed by gene 𝑦. Unlike X, protein Y represses both 𝑦 and 𝑥 simultaneously (Figure 17b) and brings about a decline in the concentration of X.

**Figure 18:**
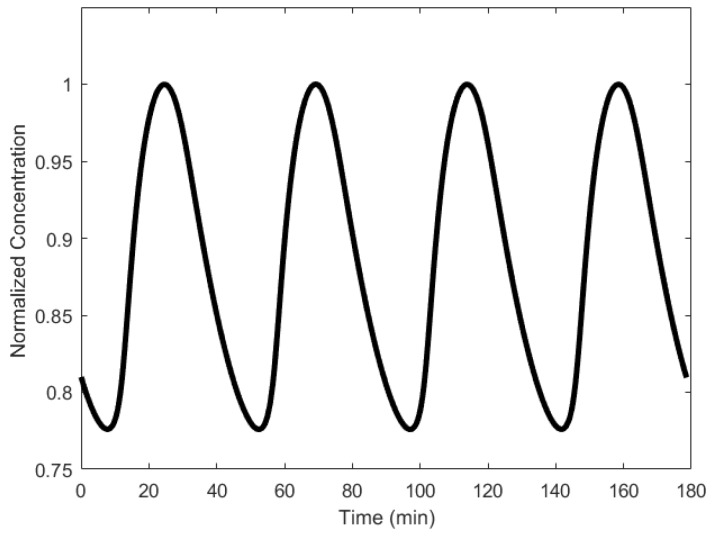
Oscillations in the concentration of *x* by the dual feedback oscillator.

Oscillations were generated with periods of about 44 min (Figure 18). In wet lab experiments, periods of approximately 40 min have been observed, suggesting a good relationship between the model and the experiment [22]. The dual feedback oscillator is extremely robust owing to the positive and negative feedback loops—more than 99% of the engineered cells exhibited oscillations successfully [22]. Unlike the Goodwin oscillator, the dual feedback oscillator also showed varying periods in the range of 13–58 min when the ITPG concentration was varied [22], indicating tunability.

#### 2.2.7. Fussenegger Oscillator

**Figure 19:**
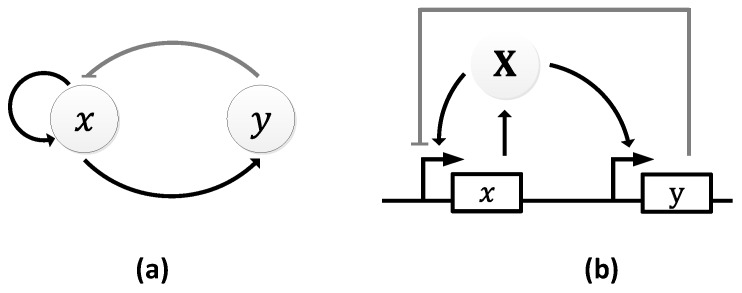
Fussenegger oscillator: (**a**) symbolic representation (**b**) schematic representation depicting the internal pathways. The grey colored lines represent the hybridization path.

The Fussenegger oscillator is a synthetic mammalian oscillator based on an autoregulated sense-antisense transcription control circuit encoding a positive and a time-delayed negative feedback loop, enabling autonomous, self-sustained oscillatory gene expression [23]. The sense strand of RNA (or antisense strand of DNA) is the strand used for the translation of a protein (or transcription of the mRNA), whereas the antisense strand of RNA (or sense strand of DNA) does nothing. However, when the sense and antisense strands of RNA form a complex just like a double helix DNA, translation can be inhibited. This is the underlying principle of the negative feedback of the Fussenegger oscillator. The Fussenegger oscillator comprises two genes, 𝑥 and 𝑦, one of which involves both sense and antisense transcription (Figure 19a). The sense transcript of 𝑥 is translated, the resulting protein X feeding back to itself by promoting transcription and activating the second gene, 𝑦 (Figure 19b). In a first for a synthetic genetic network, this second gene activates the antisense transcription of the first gene; the transcript is not translated, instead it hybridizes with the sense transcript, repressing the production of X at translation [32].

**Figure 20:**
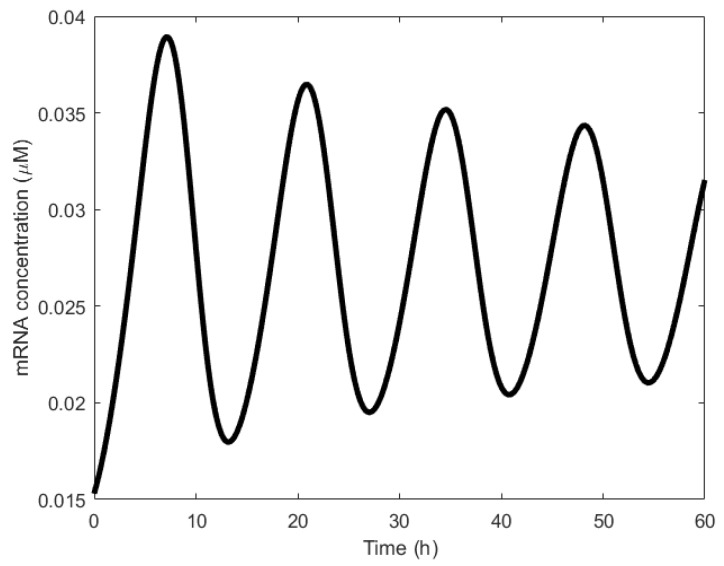
Oscillations obtained by the Fussenegger oscillator.

The Fussenegger oscillator has one of the highest periods obtained from a synthetic oscillator, averaging in the order of hours (~15 h), as shown in Figure 20. On the contrary, wet lab experiments show average periods ranging in the order of minutes (170 ± 71 min), far less that the simulated oscillations. To match the simulation results to those of the experimental results, a refined mathematical model (Equations (1.1) and (1.5) were replaced by Equations (1.21) and (1.22), respectively [23]) with estimated parameter values (see [23], supplementary materials) was designed and matching results were obtained (see Figure 4c [23]). An alternative oscillator was developed in [111] to make the first-generation oscillator [23] insensitive to component fluctuations. This resulted in a low-frequency oscillator.

#### 2.2.8. miRNA-Regulated Oscillator

**Figure 21:**
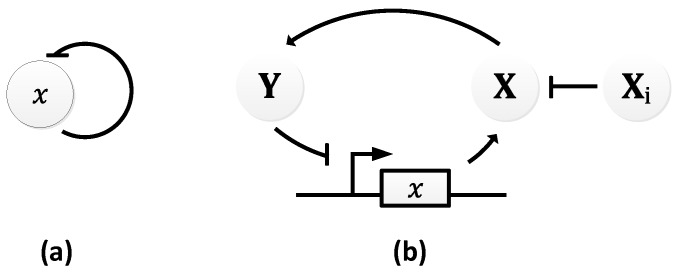
miRNA-regulated oscillator: (**a**) symbolic representation; (**b**) schematic representation depicting the internal pathways.

As the name suggest, a miRNA-regulated oscillator is one whose oscillations are regulated by miRNAs (microRNAs) where the miRNA regulation is coupled with feedback loops—either positive or negative or both. miRNA is an RNA which is not translated into a protein but instead, through base-pairing with a target mRNA, leads to post-transcriptional/translational repression, thereby inhibiting protein generation [112,113]. Based on this feature of miRNA, an oscillator model was proposed by Gerard and Novak [25]. The model rests on a negative feedback loop where mRNA (X) encodes the synthesis of a protein (Y), which acts as the repressor. The latter represses the synthesis of messenger RNA. By forming an inhibitory complex with mRNA, miRNA (X_i_) inhibits the expression of the protein (Figure 21b).

**Figure 22:**
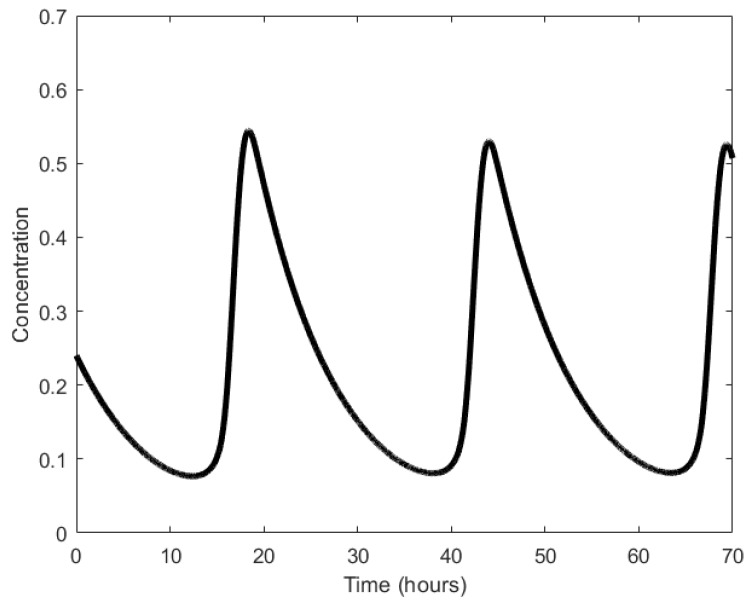
Oscillations formed in the concentration of the protein by the miRNA-regulated oscillator.

This model resembles the Goodwin oscillator but with the addition of the miRNA process (Figure 21a). In fact, the additional process introduces a larger delay which relaxes the need for an unrealistically large Hill coefficient value (n > 8) to generate the oscillations (at least eight molecules of repressors must bind in a complex to be able to repress the expression of the gene [25]). As shown in Figure 22, the oscillations are formed with n = 4, which is half that of required by the original Goodwin oscillator.

#### 2.2.9. Displacillator

**Figure 23:**
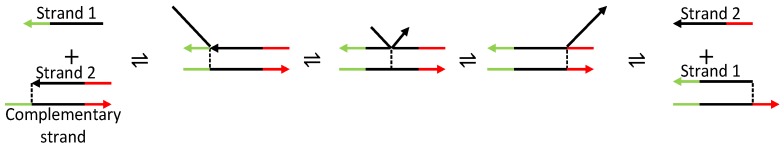
Illustration of a DNA strand displacement between two strands that share the same sequence (black line), allowing them to bind the same complementary strand.

Unlike the aforementioned oscillators, the displacillator [114] is based on a molecular interaction between DNA strands called *strand displacement* [115]. Fundamentally, a strand displacement interaction involves two DNA strands that compete with each other to bind to the same complementary strand. As shown in Figure 23, strand 1 competes with strand 2 to bind to the complementary strand resulting in the displacement of strand 2. The displacement can occur in the reverse direction where strand 2 displaces strand 1.

**Figure 24:**
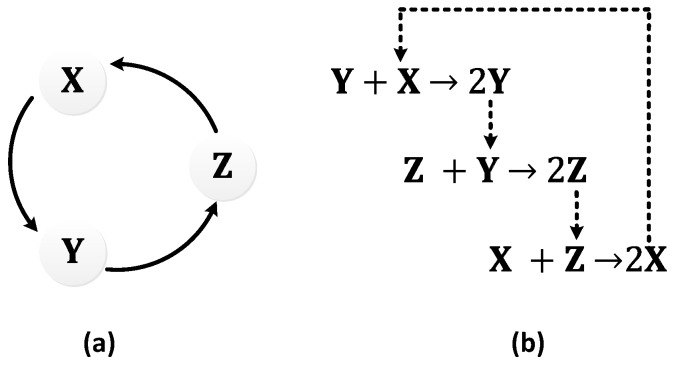
Displacillator: (**a**) symbolic representation; (**b**) schematic representation of the cascading strand displacement interactions.

**Figure 25:**
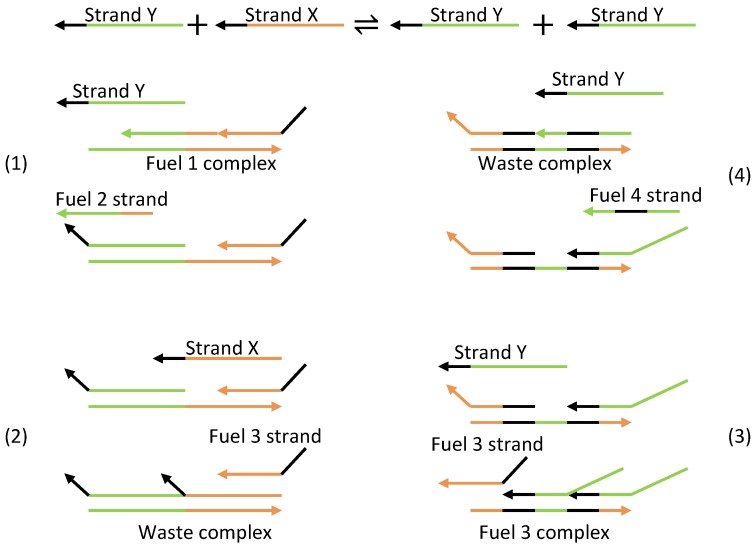
Strand displacement implementation illustrating the first part of the cascade. (**1**) Strand Y binds to the Fuel 1 complex through strand displacement. (**2**) Strand X also binds to the Fuel 1 complex through strand displacement, releasing the Fuel 3 strand. (**3**) The Fuel 3 strand binds to the Fuel 3 complex through strand displacement, releasing the first Y strand Y from the complex. (**4**) The Fuel 4 strand also binds to the Fuel 3 complex through strand displacement, releasing the second Y strand.

**Figure 26:**
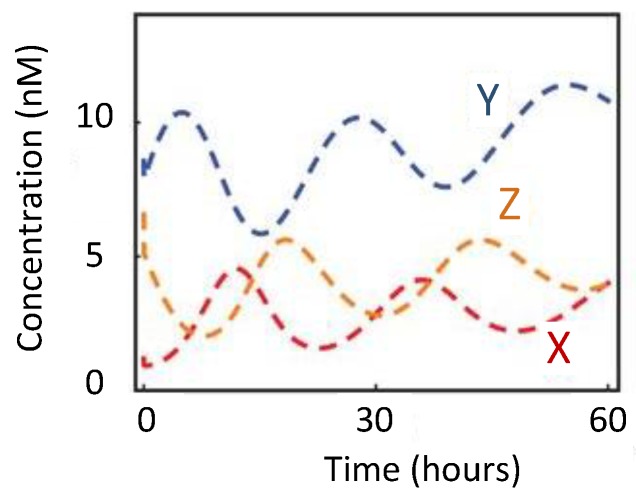
Oscillations observed in individual strands of the displacillator (see [114], supplementary materials). From Niranjan Srinivas, James Parkin, Georg Seelig, Erik Winfree, David Soloveichik, Enzyme-free nucleic acid dynamical systems, Science, 358, December, 2017. Reprinted with permission from AAAS.

Based on the aforementioned mechanism, three strands or molecules become interlinked with each other (Figure 24a). The displacillator involves several strand displacement interactions that are cascaded, as shown in Figure 24b. In the first reaction of the cascade, strand (or molecule) X is consumed to generate more Y, which is further consumed in the second reaction to generate more Z, and finally, Z is consumed in the third reaction to generate more X, completing the cascading loop. However, this figure is an oversimplification of the reaction, as the strands on the left side of the reaction do not directly interact with one another. Instead, additional DNA complexes called fuels are used to facilitate the reaction. These fuel complexes undergo a two-step stand displacement on each side of the reaction aided by the strands themselves or other fuel strands, as depicted in Figure 25. The oscillations observed in the displacillator are shown in Figure 26.

Unlike their natural counterparts, the synthetic oscillators described above could be embedded within a nanomachine allowing constant availability of the timing information to a nanomachine and its communication modules. This is the main advantage of such systems as it allows network-related services such as data aggregation/fusion, scheduling techniques, time-divided channel access, etc. to be realized. However, it may require the oscillators to be synchronized relatively, meaning each oscillator may synchronize with other oscillators using the information about clock drift and clock offset or to be synchronized absolutely, meaning the oscillators synchronize to a global time.

In Table 2, we summarize the characteristics of the synthetic oscillators and list them in the ascending order of oscillation frequency. In terms of the number of cells that successfully oscillate, the dual feedback oscillator can achieve a very high success rate, with 99% of the cells achieving oscillations. The metabolator comes in second with a success rate of 60%. Common to both these oscillators are the positive and negative feedback loops inherent in the models. With a success rate below 50%, the repressilator is third and interestingly, it does not involve a positive feedback loop. While there could be other intrinsic factors such as the values of the system design parameters, having both positive and negative feedback loops seems to have a positive impact on the success rate.

### 2.3. Oscillators Specific to the Nanonetwork

In Section 2.2, we examined and reviewed the synthetic oscillators that were strictly developed by biologists and, in a way, can be thought of as endeavors to identify new methods of engineering systems at the molecular level. Having said that, the nanonetwork research community have also proposed oscillatory systems whose mechanisms are largely inspired by natural oscillators or mechanisms to perform and provide functions pertaining to communication devices. We discuss them in the subsequent paragraphs.

#### 2.3.1. Moore Oscillator

**Figure 27:**
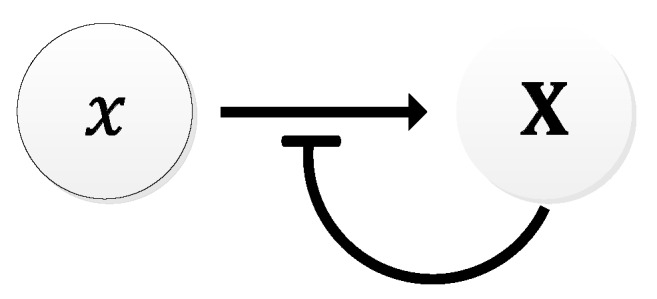
Schematic diagram of the Moore oscillator.

**Figure 28:**
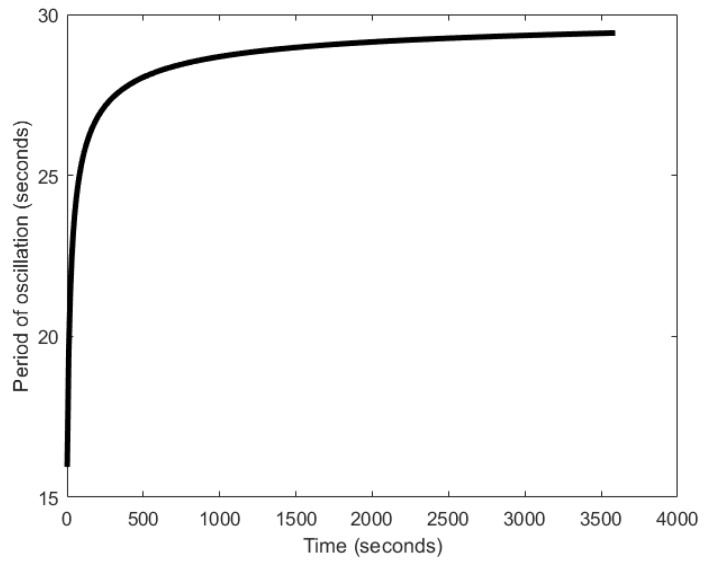
Time evolution of the period of oscillation obtained by Moore oscillator.

In [29,116], the authors proposed a system with auto-inhibitory molecules to generate oscillations. The mechanism that drives the oscillator is based on a naturally occurring phenomenon, wherein, the coupling of two feedback signals, excitatory and delayed inhibitory, results in oscillations [117]. The excitatory path is formed when the type 𝑥 molecules chemically react to release a pulse of type X molecules. Type X molecules are of inhibitory nature and therefore, once released into the environment, they begin to inhibit the type 𝑥 from further generation of X pulses, forming delayed inhibitory feedback (Figure 27). Then, as the type X molecules disperse into the environment, their concentration around 𝑥 decreases. Beyond a certain concertation level, they can no longer inhibit 𝑥, and therefore, 𝑥 can then release the next pulse of type X molecules. The attainment of the lower threshold marks the completion of one oscillation cycle. The quick release and slow dispersion gives the oscillation a relaxation oscillator attribute. These processes keep repeating, forming oscillations. The period of oscillation can range between 15–25 s (Figure 28). In some ways, the Moore oscillator resembles the Goodwin oscillator [100,101].

#### 2.3.2. Akgül Oscillator

**Figure 29:**
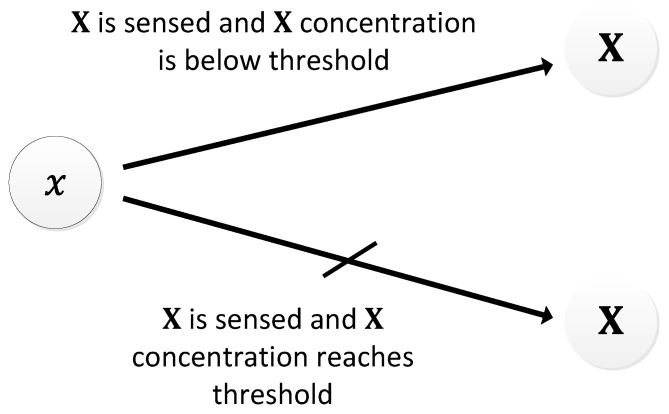
Schematic diagram of the Akgül oscillator. The arrow with the crossed line indicates that X is not generated.

**Figure 30:**
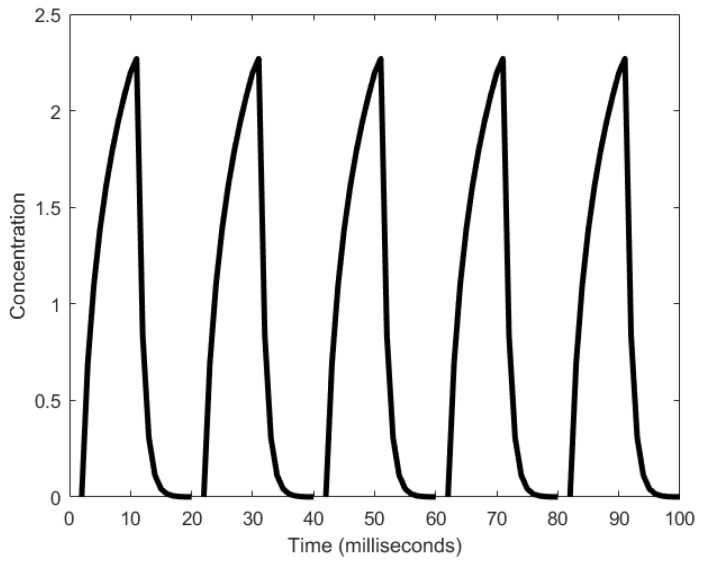
Global oscillations generated in the concentration of the auto-inducing molecule by the Akgül oscillator.

In another study [30], the authors proposed an oscillatory system that is regulated by auto-inducing molecules. Besides the fact that the auto-inducers facilitate their own generation, the proposed system works at the network level. The basis of this oscillator is also from a naturally occurring phenomenon known as *quorum sensing* [118]. When a nanomachine senses the auto-inducers in the environment, it immediately releases the auto-inducer. The released auto-inducing molecules propagate via diffusion to other nanomachines, causing them to release more auto-inducing molecules. Every nanomachine performs this type of chain reaction process until the concentration of the auto-inducer reaches a certain upper threshold. Once the concentration reaches the upper threshold, the nanomachine halts the release of the auto-inducers, and this action causes a decrease in the concentration of the auto-inducers. As the concentration falls to a lower threshold, the nanomachines begin to release the auto-inducers, causing a rise in the concentration again (Figure 29). The resultant periodic increase and decrease in the concentration of the auto-inducers constitutes the oscillations. An oscillation period of about 10 s can be achieved, as shown in Figure 30.

#### 2.3.3. Shitiri Oscillator

**Figure 31:**
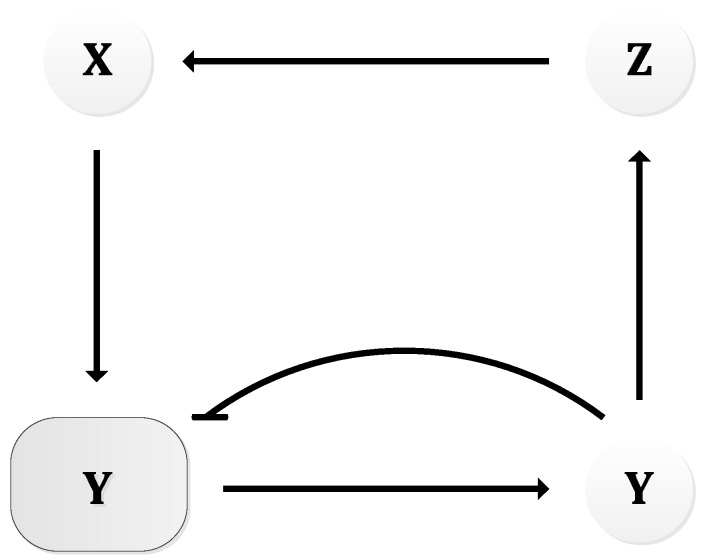
Schematic diagram of the Shitiri oscillator. The rectangular box represents the storage unit of Y.

**Figure 32:**
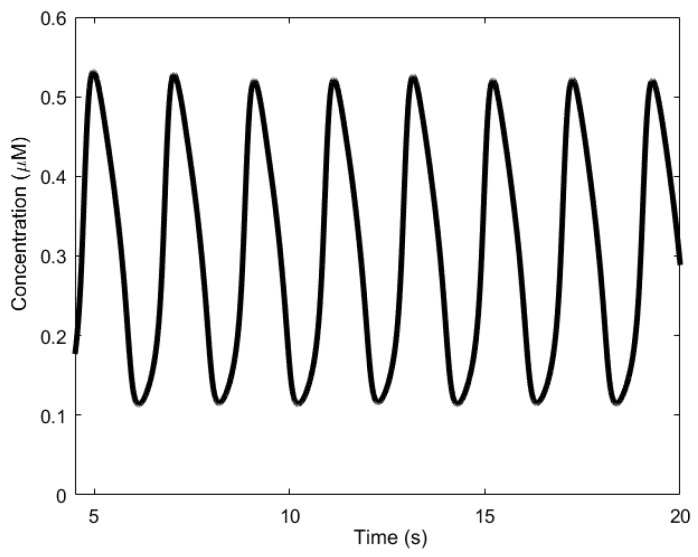
Oscillations in the concentration of the Y molecule generated by the Shitiri oscillator.

A more recent study [31] has proposed a system that uses excitatory molecules to generate oscillations with a higher frequency. It draws inspiration from Ca^2+^ oscillators [85]. It employs three types of excitatory molecules (X, Y, and Z) that work in tandem to generate oscillatory patterns in the concentration of molecule Y. In addition, the system consists of an internal store of Y molecules. When the X molecules bind to the biphasic receptors that are in the internal store [79], the Y molecules are released, increasing the concentration of Y outside the store. As Y increases, this causes the receptors to shut, thereby stopping any further outflow of Y molecules. This step, along with the Y molecules being pumped back into the store, forms the negative feedback loop of the system. At this stage, the concentrations of the X and Y molecules decline. However, during the increase in Y molecules, the Y molecules trigger the reproduction of X molecules by chemically reacting with the Z molecules. This causes the concentration of X molecules to increase, and the whole cycle begins again. Figure 31 illustrates the process. A period of oscillation of about 2 s can be achieved (Figure 32).

These oscillators have been designed with consideration of the nanonetwork and its applications. Similar to the synthetic oscillators presented in Section 2.2, the aforementioned oscillators could be embedded into a nanomachine. In addition to the communication-related services mentioned in Section 2.2, the aforementioned oscillators could allow the correct decoding of signals or even allow faster data rates. However, what they lack is laboratory validation.

Table 3 summarizes the characteristics of the aforementioned oscillators and, as one would expect, none of them share the same process for generating the oscillations. Interestingly, these oscillators have higher frequencies compared to those listed in Table 2, at least in principle.

Table 4 provides a qualitative comparison of the synthetic oscillators presented in this study. *Robustness* is a measure of the number of oscillators in a nanonetwork that achieve oscillations. Simply put, it is the ability of an oscillator to overcome the molecular noise (more details in Section 3.1) and produce oscillations as desired. *Tunability* is the ability of an oscillator to modify the frequency of oscillations when a specific trigger is applied. This feature enables the rectification of clock skew, if it exists, between the oscillators. From Table 4, we can infer that the combination of a positive and a negative feedback loop improves the robustness. When more than one of these combinations exists, the robustness is very high, as in the case of the dual feedback oscillator. However, we can also infer that when more than one negative feedback loop is combined with sufficient delay, improvements in the robustness are observed, as in the case of the repressilator. From these inferences, we can cautiously acquire some insight into the robustness of the Moore and Shitiri oscillators—i.e., they are highly robust. With regard to tunability, less than half of previous works analyzed tunability in their experiments, with two-thirds reporting tunability. Although not shown in the Table 4, natural oscillators, in general, are tunable. With that being said, Moore, Akgül, and Shitiri oscillators should be able to exhibit tunability as they are based on natural oscillators. To some degree, the Shitiri oscillator has been shown to modify the oscillations in response to changes in the initial concentrations of the stimuli. Table 5 presents a summary of the advantages, disadvantages, types (manner in which the oscillators convey the time information), and frequencies of the biological oscillators.

## 3. Open Research Issues

### 3.1. Noise

Gene expression, which is one of the main processes that is responsible for the oscillations in the synthetic oscillators discussed in Section 2.2, involves a series of biochemical processes that are discrete in nature, causing *molecular noise* [119]. This noise is fundamentally made up of two components—intrinsic and extrinsic noises. Intrinsic noises, also referred to as the inherent stochasticity, are the variations in the rate of a particular gene’s expression among identical cells due to microscopic events that govern which reaction occurs and in what order [119]. On the other hand, extrinsic noises are the fluctuations in the output of the gene due to the fluctuations in the amount or activity of the regulatory molecules (proteins and polymerases). This is because the expression of each gene is controlled by the concentrations, states, and locations of the regulatory molecules [119]. In fact, molecular noise is inversely proportional to the √(number of molecules) [120].

To take into account the effects of molecular noise on the oscillatory behavior, stochastic numerical models [121,122] are preferred over deterministic differential equation models. Nonetheless, deterministic models provide good approximations for oscillatory behavior when molecules in quantities greater than hundred are present in the oscillatory mechanism [123,124].

The molecular noise can be controlled to a lower level by producing fewer proteins from numerous mRNA (low translation rate and high transcription rate) or by producing fewer mRNAs (low transcription rate) but this comes with an energy cost [125]. A further reduction in noise can be achieved using a negatively auto-regulated transcription system to form a negative loop [126], echoing why the negative feedback loops are so vital in generating sustained oscillations (as seen in Section 2). Using a larger amount of molecules can also reduce the noise levels [120]. However, while the molecular noise may be reduced by using a large number of molecules, the interference between different species of molecules may increase. Therefore, a tradeoff between noise and interference arises. Finding the optimal number of molecules that a system can handle without significant noise and interference remains an open research challenge.

### 3.2. Design

The oscillators discussed in Section 2.3 lack validation in wet lab experiments. While mathematical models have been very effective in capturing the complex physical behaviors of the oscillatory systems and realizing them in computer simulations, it would be highly beneficial for such systems to be further validated in wet lab experiments. As seen in the case of the Fussenegger oscillator, a significant difference in the period of oscillation between the results obtained in from computer and wet lab experiments was obtained. However, a minor revision to the mathematical model corrected the difference. On the contrary, a very good agreement between the mathematical model and the wet lab experiments was observed for the Atkinson oscillator. From the latter, it is safe to infer that with mathematical models, if done correctly, it is possible to replicate results exactly as one would obtain in the wet lab experiments. Such precise modelling can in fact reduce the requirement of validating the model in wet lab experiments, at least in the developmental stages, as it should be noted that the time and cost involved in wet lab experiments is higher than in computer simulations.

One way to ensure that a model will indeed oscillate is to adhere to specific design guidelines, such as those presented by Novak and Tyson [24]. Four fundamental requirements for the design of an oscillator are negative feedback, time delay, sufficient non-linearity of reaction kinetics, and proper balancing of time scales of opposing chemical reactions. An oscillatory system should be able to return to its starting point, which is achieved through negative feedback. To ensure the chemical reactions involved in the oscillations do not settle at a stable steady state (no oscillations), the negative feedback signal must be sufficiently delayed in time, and thereby, obtain sustained oscillations. Non-linearity is essential to destabilize the steady state and therefore, the kinetic rate laws of the reaction mechanism must be sufficiently “nonlinear”. The reactions that produce and consume the interacting chemical species must occur on appropriate timescales that permit the network to generate oscillations. Such guidelines, therefore, lay the framework for new and novel oscillatory systems to be designed in principle without having to worry about the correctness of the design, as long as the requirements are met.

Arguably, a tradeoff between time, cost, and ease of rectification and validation arises. However, it should also be noted that both techniques complement, rather than oppose, each other. Therefore, as much as wet lab experiments allow for practical realizations, it would be sufficiently enough to fully develop and test an oscillatory system using computer simulations as mistakes in the model can be corrected much easier than in the wet lab.

### 3.3. Sustainability

Molecules are associated with a finite lifetime. They undergo degradation over the course of time and as such, oscillations cease to exist. This makes the selection of molecules of utmost importance. Two methods can be used to enhance the lifetime of the molecules and guarantee the sustainability of an oscillator. First, molecules that have very long lifetimes or more specifically, longer half-lives must be carefully selected, such as Ca^2+^ which can have a half-life of up to months or years [127]. Second, the degraded molecules must be replaced frequently with newly synthesized molecules of the same kind. This mechanism is commonly found, as seen in synthetic oscillators, where the periodic process of transcription and translation generates new molecules of mRNA and protein/metabolites, respectively.

To have an energy efficient system, it would be desirable to use the first method. However, this requires some sort of storage facility, such as the endoplasmic reticulum found in calcium oscillators, to store, release, and absorb back a particular molecule—a higher complexity in the design. On the contrary, the second method does not have the need for a storage facility. However, due to the constant synthesis of new molecules, a sufficient amount of ATP needs to be available at all times to carry out the synthesis—a higher or constant energy demand. This creates, to some degree, a tradeoff between energy and complexity.

### 3.4. Adoption and Implementation to Nanonetworks

To date, no experimentation validation has been found that shows how an actual transceiver-like nanomachine works and interacts with other nanomachines or within its communication modules. This leaves us to cautiously step into the unknown future in the actual realizations of such nanomachines. Nonetheless, the recent developments in nanotechnology assure us a better future. Considering this, we outline here a couple of key implementation issues.

#### 3.4.1 Interfacing

As pointed out in Section 3.1, one of the pressing issues is to provide an interface between a nanomachine and an oscillator. This could be achieved through a special unit, namely the *biomolecule detector*, to read the changes in the oscillations and convey the message to a nanomachine. Such interfacing mainly applies for oscillators that are not embedded within a nanomachine but can also be extended to those oscillators that are be embedded within a nanomachine. For the ones embedded in a nanomachine, the challenge lies in passing the information about time in a secure and fast manner to every communication module. Molecular motors, which are proteins or protein complexes that transform chemical energy into mechanical work, could act as potential carriers of timing information [1]. However, investigations into the engineering of biological oscillators to molecular communication systems and specifics into how these technologies can be incorporated for potential applications of nanonetworks still remain research challenges.

#### 3.4.2. Matched Oscillators

As pointed out in Section 3.2, another pressing issue is the need to synchronize the oscillators. We envision two methods to achieve that goal—internal and external. The internal method refers to cases where a special module, namely the *period checker*, ensures a constant desired period of oscillations (or zero clock drift). Equipped with biological sensors, the period checker routinely checks the period between two consecutive oscillations. If deviations from the desired period are observed, then it will release a specific molecule to counteract the deviation. Such modifications to the period have been observed in calcium oscillators [128]. However, this increases the design complexity and may not be able to handle clock offsets. The external method refers to cases where algorithms and protocols are designed to estimate the clock drifts and the clock offsets. These methods already exist in traditional communication networks and are beginning to emerge in the literature for nanonetworks [29,129,130]. Such methods are much simpler to implement but could be prone to channel variation and may have high energy requirements. Therefore, suitable protocols still need to be developed.

## 4. Conclusions

Oscillators essentially act as “heartbeats” and provide the necessary timing information to electronic devices [14]. On that note, it is pertinent for nanomachines to be embedded with oscillators as nanomachines are envisioned to perform functions such as periodic measurements, coordinated activity, and scheduled transmissions, etc., all of which are required to be regulated with precise timing. In a tutorial fashion, we have presented the recent advancements in biological oscillators. We have broadly classified biological oscillators into two groups—natural and synthetic. As expected, in most cases, synthetic oscillators have much simpler mechanisms than their natural counterparts. Frequencies greater than 1.67 mHz have been obtained in-vivo for synthetic oscillators, and frequencies of up to 1000 mHz have been obtained in natural oscillators. Moreover, in principle, synthetic oscillators could achieve up to 500 mHz and it remains to be seen if the same can be achieved through wet lab experiments.

With the necessary framework [24] set up to act as a guide in the design of the oscillators, we outlined the issues in the physical aspects with regard to molecular noise, the precision of models, and the lifetime of molecules. To counteract molecular noise without incurring a higher energy cost, there remains a tradeoff between the number of molecules and the tolerable interference between different molecules. Another challenge that remains is the precision of the models in comparison to wet lab experiments. While computer models are easier to correct and require less time to generate results, adequate care is needed to ensure the precision of the model. Arguably, a tradeoff between time, cost, and ease of rectification and validation arises. Finally, the effects of the degradation of the molecules employed in the oscillatory system has to be sufficiently long, and similarly, the lifetime of the network or suitable molecule replacement mechanisms, such as synthesis, should be incorporated. This leads to a tradeoff between energy and complexity. We believe that these challenges and issues will serve as a guide for future research development in biological oscillators.

Additionally, we provided discussions on the issues related to the communication aspects of the oscillators. Two key issues—namely interfacing and matched oscillators—were identified and presented as potential future research directions.

## Figures and Tables

**Table 1:** Overview of natural oscillators.

Oscillator	Year of Discovery	Cell/Organism	Frequency	Process Regulating the Oscillations
Circadian oscillator	1729 [65]	Unicellular and multicellular	11.57 μHz	Transcription regulation
Mitotic oscillator	1974 [92]	Eukaryotic cells	11.57 μHz to 1.7 mHz	Enzyme regulation
Glycolytic oscillator	1965 [35]	Yeast cells; heart cells; muscle extracts; Pancreatic beta cells;	0.3–16.7 mHz	Enzyme regulation
cAMP oscillator	1974 [50]	Dictyostelium discoideum	1.7–3.33 mHz	Receptor-enzyme interactions
Calcium oscillator	1985 [76]	Variety of cells	16.7 mHz to 1 Hz	Transport between cells and their environment or between various intracellular compartments

**Table 2:** Overview of synthetic oscillators.

Oscillator	Year	Frequency: Theoretical/Experimental	Oscillations Observed	Process Involved in the Oscillations
miRNA-regulated oscillator	2013 [25]	11.57 μHz/n.a	n.a.	Post-transcription regulation
Displacillator	2017 [114]	0.0139 mHz/0.0139 mHz	n.a.	Strand displacement
Fussenegger oscillator	2009 [23]	0.019 mHz/0.098 ± 0.23 mHz	n.a.	Post-transcription regulation
Atkinson oscillator	2003 [106]	0.028 mHz/0.02 mHz	n.a.	Transcription regulation
Repressilator	2000 [21]	0.11 mHz/0.1 ± 0.42 mHz	40%	Transcription regulation
Dual feedback oscillator	2008 [22]	0.38 mHz/0.42 mHz	99%	Transcription regulation
Hasty oscillator	2001 [107]	(0.38–2.08) mHz/n.a.	n.a.	Transcription regulation
Metabolator	2005 [108]	0.42 mHz/1.67 ± 0.37 mHz	60%	Metabolic and transcription regulation
Goodwin oscillator	1963 [100]	0.56 mHz/n.a.	n.a.	Transcription regulation

**Table 3:** Overview of oscillators specific to the nanonetwork.

Oscillator	Year	Frequency: Theoretical/Experimental	Oscillations Observed	Process Involved in the Oscillations
Moore oscillator	2013 [29]	50 mHz–mHz/n.a.	n.a.	Auto-inhibition
Akgül oscillator	2014 [30]	100 mHz/n.a.	n.a.	Auto-inducer
Shitiri oscillator	2016 [31]	500 mHz/n.a.	n.a.	Molecule-receptor interactions

**Table 4:** Qualitative comparisons of the synthetic oscillators.

Oscillator	Feedback Loops	Model-to-Wet Lab Agreement	Robustness	Tunability	Oscillations
Goodwin oscillator	One self-(−)	Bad	Low	No	Sustained
Repressilator	Three (−)	Good	Medium	n.a.	Sustained
Atkinson oscillator	One self-(−) and one (−)	Excellent	n.a.	n.a.	Damped
Hasty oscillator	One self-(±), one (±), and one (−)	n.a.	n.a.	n.a.	Sustained
Metabolator	One (−) and one (+)	Good	Medium	n.a.	Sustained
Dual feedback oscillator	One (−), one self-(−), one (+), and one self-(+)	Good	High	Yes	Sustained
Fussenegger oscillator	One self-(−) and one (−)	Good after revision	n.a.	n.a.	Damped
miRNA-regulated oscillator	One self-(−) and one coupled (−)	n.a.	n.a.	n.a.	Sustained
Displacillator	Three (+)	Good	n.a.	Yes	Damped
Moore oscillator	Two self-(−) and two (−)	n.a.	n.a.	n.a.	Sustained
Akgül oscillator	One self-(+)	n.a.	n.a.	n.a.	Sustained
Shitiri oscillator	Two self-(−) and one (+)	n.a.	n.a.	Yes	Sustained

**Table 5:** Summary of biological oscillators.

Oscillator	Advantages	Disadvantages	Type	Frequencies
Natural	Not embedded into a nanomachine Simpler to implement	Close proximity Additional interfacing	Broadcasting only	Low
Synthetic	Embedded into a nanomachine Allows more functionalities	Synchronization required	Peer-to-peer or broadcasting	Low–medium
Synthetic nanonetwork	Embedded into a nanomachine Allows more functionalities	Synchronization required Lacks laboratory validation	Peer-to-peer or broadcasting	Medium–high
